# Brain markers of neurodegeneration in sepsis survivor rats

**DOI:** 10.1186/cc12986

**Published:** 2013-11-05

**Authors:** Larissa de Souza Constantino, Cristiane Damiani Tomasi, Matheus Pasquali, Samantha Pereira Miguel, João Paulo Almeida dos Santos, Francieli Vuolo, Clarissa Martinelo Comim, Fabrícia Petronilho, João Quevedo, Daniel Pens Gelain, José Cláudio Fonseca Moreira, Felipe Dal-Pizzol

**Affiliations:** 1 University of the Extreme-South Catarinense, Criciúma, Brazil; 2 Federal University of the Rio Grande do Sul (UFRGS), Porto Alegre, Brazil; 3 University of Southern Santa Catarina, Tubarão, Brazil

## Background

Several preclinical and clinical reports indicate a significant role for systemic inflammation in chronic neurodegenerative diseases [1], with commitment of different brain regions. Several studies have demonstrated hippocampal atrophy, EEG changes [2], profound glial activation, the generation of nitric oxide and changes in expression of mediator apoptosis [3]. The release of these mediators and oxidative stress occur mainly in acute phase inflammation in sepsis survivor rats and are associated with long-term cognitive impairment [4]. These cognitive deficits have been associated with decreased quality of life and increased long-term morbidity. Some of these alterations resembled the pathophysiological mechanisms of neurodegenerative diseases. For this reason, we analyzed parameters related to neurodegeneration in rats that survived sepsis, and their relation to cognitive dysfunction.

## Materials and methods

Wistar rats were subjected to sepsis by cecal ligation and puncture and 30 days after surgery the hippocampus and prefrontal cortex were isolated just after cognitive evaluation by the inhibitory avoidance test. The immunocontent of β-amyloid peptide (Aβ), receptor for advanced glycation endproducts (RAGE) and synaptophysin were analyzed by western blot.

## Results

Aβ was increased in septic animals in the hippocampus, but not in the prefrontal cortex. RAGE was upregulated in both structures after sepsis, and the immunocontent of synaptophysin was decreased only in the prefrontal, and inversely correlated to Aβ levels. Prefrontal levels of synaptophysin correlated with performance in the inhibitory avoidance.

## Conclusions

The brain from sepsis survivor animals presented several markers of neurodegeneration, and inhibitory avoidance test performance seems to be dependent on the levels at some of these markers.
